# Separation of intrinsically magnetic cells using magnetic filters

**DOI:** 10.1016/j.seppur.2025.132994

**Published:** 2025-04-18

**Authors:** Jacob Strayer, Hyeon Choe, Xian Wu, Poornima Ramesh Iyer, Jenifer Gómez-Pastora, Will Moorman, Joseph Cefaratti, Alec West, Kristina Landes, Payal C. Desai, Andre F. Palmer, Maciej Zborowski, Jeffrey J. Chalmers

**Affiliations:** a William G. Lowrie Department of Chemical and Biomolecular Engineering, The Ohio State University, Columbus, OH, USA; b Department of Chemical Engineering, Texas Tech University, Lubbock, TX, USA; c College of Arts and Sciences, The Ohio State University, Columbus, OH, USA; d Wexner Medical Center, The Ohio State University, Columbus, OH, USA; e Levine Cancer Institute, Atrium Health, Charlotte, NC, USA; f Department of Biomedical Engineering, Lerner Research Institute, Cleveland, OH, USA; g Department of Biomedical Engineering, Cleveland Clinic, Cleveland, OH, USA

**Keywords:** Red blood cells, Magnetic separation, Packed column separation, High gradient magnetic separation, Computational simulation, Numerical modeling

## Abstract

Cell separation is a common industrial, clinical, and laboratory technique that is typically accomplished via centrifugation. However other technologies for cell separation are employed when the samples need to be separated based on biological/physical properties apart from cell density. Magnetism is a technique that can be employed to separate biological entities. Cellular magnetism can be intrinsic (i.e., paramagnetic), such is the case for deoxygenated red blood cells (RBCs) and certain cancer cells that exhibit abnormal iron metabolism (e.g., glioblastoma), or this magnetism can be artificially induced based on magnetic particle labeling. In this work, we investigate magnetic filtration using commercial, high gradient magnetic separation (HGMS) packed columns for the separation of intrinsically magnetic RBCs. Specifically, flow rate, cell concentration, and external field strength were investigated using Miltenyi LS columns as magnetic filters. A generalized model for magnetic filtration was applied and used to investigate potential scale up of our RBC magnetic filtration process. COMSOL Multiphysics numerical simulations were performed to quantify the effect of the external field strength on the local magnetic energy gradient generated inside the ferromagnetic packed column. It was found that varying the externally supplied magnetic field from 0.1 to 1 T produces a non-linear increase in the magnetic energy gradient local to the ferromagnetic packing structure in the separator. Finally, the magnetic separator was used to demonstrate the capability of a binary separation of an artificially produced mixture of methemoglobin (metHb) containing RBCs (paramagnetic) from non-oxidized ferrous oxyhemoglobin (oxyHb) containing RBCs (diamagnetic). It was shown that 99 % of the magnetically isolated cells had a magnetic signature indicative of metHb-RBCs. In conclusion, we demonstrated the application of a magnetic filtration model for RBC separation and its utility to study the process variable effects on the separation’s performance.

## 1. Introduction

Body fluids and tissues are a source of information about an individual’s health. A crucial step for this in-depth analysis is the separation of target cells from complex biological samples, such as blood or tissue components, with minimal contamination from the non-target cells [1,2]. Hence, many separation technologies have been developed to isolate cells from various biological samples, such as blood. These methods fall into three categories: physical approaches, which use properties such as size or density; surface or affinity-based approaches, which use fluorescently tagged or magnetically tagged affinity molecules that target specific cell surface markers; and combinations of the two approaches [3–9]. Given the significance of affinity-based identification and separation approaches, the majority of the research and commercial products have focused on isolating specific cells from the blood sample [5,10–12].

The universal first step in isolating cells from a blood sample is the removal of red blood cells (RBCs), typically achieved by centrifugation [13–15] or through the selective lysis of RBCs [16–20]. However, this step is generally seen as a routine procedure, and significant effort has not been invested in optimizing the separation of RBCs, as the primary interest in most of the research and clinical settings tends to focus on the other blood components. However, neither centrifugation nor RBC lysis yields an optimal product. Centrifugation is time-consuming, is relatively expensive on larger scales, requires trained personnel, can be non-reproducible, requires multiple rounds to remove all the RBCs, and can potentially damage the RBCs due to the high shear forces generated [21–23]. On the other hand, RBC lysis with chemical buffers can damage white blood cells or the targeted rare cells and the residual components from the lysed RBCs can interfere with downstream analysis [24–26]. With these limitations in mind, it is important to develop a technique that can remove RBCs efficiently while minimizing cell damage.

A promising yet underutilized alternative to removing RBCs is magnetic separation. Traditionally, researchers have focused on imparting magnetic susceptibility to a targeted cell by binding it to a magnetic micro/nanoparticle. However, there is a growing interest in the “label-free” magnetophoretic separation of RBCs, a technique first demonstrated by Melville and co-workers in columns packed with steel wire [27]. The label-free magnetophoretic isolation of cells has been demonstrated with deoxygenated or oxidized RBCs, (typically referred to as the “met state” due to the presence of methemoglobin) [28–33], cancer cells [34], as well as other cells that had been previously incubated with different forms of iron such as macrophages [35]. It has also been suggested that magnetic separation of paramagnetic cells can be employed for the diagnosis of different disorders, such as anemia or cellular ferroptosis – a newly discovered regulated cell death mechanism based on accumulated intracellular free-iron [36,37]. Building on this concept, the principles behind magnetic cell separation can be understood through mathematical modeling, as discussed in the next section. Since RBCs are weakly paramagnetic (those with low Δχ and magnetization), High Gradient Magnetic Separation (HGMS) systems are generally employed to separate them from a blood sample. The high gradient is produced in stationary columns that are packed with ferromagnetic wires or spheres that can dehomogenizes the magnetic field, subsequently generating high magnetic field gradients in the presence of an external magnetic field [38,39].

Probably the most well-known example of an HGMS system is the magnetic-activated cell sorting (MACS) system developed and commercialized by Miltenyi Biotech (Bergisch Gladbach, Germany). When these columns are placed within an externally applied magnetic field, each ferromagnetic packing entity is magnetized, dehomogenizing the applied magnetic field within the column, and subsequently creating a very strong localized magnetic field gradient ∇B, (more than 100 Tm^−1^). When cells are passed through these systems, they experience a high magnetic force in the region adjacent to the ferromagnetic packing material, enabling their capture.

These columns have been widely used to separate different types of T cells, circulating tumor cells, and fetal or malarial red blood cells for various research applications, from basic to clinical [40,41]. The major advantages of these columns include their relatively low cost, rapid sample processing, ability to sort cells from a crude and highly concentrated biological sample, and less energy intensive since permanent magnets are used [42]. However, the primary reason why these systems are gaining popularity is that they are biologically noninvasive, since there is no physical contact between the cells and the permanent magnets, eliminating the possibility of physical damage to the cells [43]. These systems preserve the functionality of the cells, enabling further downstream cell analysis. Table 1 shows some studies on the label-free and labeled magnetic separation of RBCs from blood samples using the MACS column.

Many studies using a HGMS involved the usage of antibody-conjugated magnetic microbeads to capture the specific cells that were then separated from the other subpopulations of cells. However, these cells must be separated from the magnetic beads by physical forces or chemical methods that can compromise the cells’ functions for further downstream analysis. A solution to the removal of antibody-magnetic bead conjugates from targeted cells with a discernably different magnetic moment is to optimize label-free separation, such as the separation of RBCs. Even though a significant number of studies have been conducted on label-free separation, these studies focus on improving the separation efficiency of the columns in terms of yield and purity of the separated RBCs and, to some extent, performed downstream cell analysis. However, a major research gap in this technique is the lack of an analytical model to accurately describe the separation process and the factors influencing this process, which our study aims to start address.

This study uses a commercial MACS column to investigate the label-free separation of red blood cells (RBCs) from blood samples, focusing on factors that influence separation efficiency. The magnetization of the column’s ferromagnetic packing is affected by its elemental and crystal structure and the strength of the applied external magnetic field. The research aims to determine how increasing the magnetic field strength (B) enhances separation efficiency while examining the effects of sample flow rate and RBC concentration on capture efficiency. These studies are guided by applying a model developed by Abassov et al., a mathematical model of magnetic filtration of magnetic particles in an industrial setting [44–46]. To the best of our knowledge, this represents the first effort to integrate COMSOL simulations, magnetic filtration modeling, and experimental results to identify key factors influencing the performance of MACS columns for RBC separation.

## 2. Theory

### 2.1. Mathematics describing magnetic cell separation

Fundamentally, cells, either labeled with magnetic particles, or based on their intrinsic magnetic susceptibility, are separated via an externally applied magnetic field, that generates a magnetic force, $F_{\text{mag}}$, acting on the cells. This force can be presented as

(1) $$F_{\text{mag}}=M_{\text{cell}}V\nabla B$$

where ${\text{M}}_{\text{cell}}$ is the magnetization of the cell, V is the volume of the cell, $\mu_{0}$ is the permeability of free space, and ∇B is the magnetic field gradient. In keeping with the standard physical notation, the vector quantities are denoted by bold, normal font, the scalar quantities are italicized, and the straight brackets indicate the magnitude of a vector (such as B=|B|). The nabla symbol, ∇=[∂/∂x,∂/∂y,∂/∂z] denotes a 3D gradient operator acting on a scalar, and $B•\nabla =B_{x}(\partial /\partial x)+B_{y}(\partial /\partial y)+B_{z}(\partial /\partial z)$ indicates the gradient operator acting in the direction of the field B.

Magnetic nano- or microparticles can also be used as conjugated “tags”, such as when surface markers on nucleated blood cells are targeted with antibody coated nanoparticle labels (*i.e*. MACS reagents); the tags typically consist of ${\text{Fe}}_{\text{x}}{\text{O}}_{\text{y}}$ compounds that saturate, with respect to magnetism, at relatively low values of magnetic fields (i.e. on the order 0.1 Tesla). In these situations, Eq. (1) can be represented by:

(2) $$F_{\text{mag}}=nM_{\text{sat}}V_{\text{tag}}\nabla B$$

Where n is the number of nano- or microparticles that are attached to the cell, and ${\text{M}}_{\text{sat}}$ is the saturation magnetization of the micro/nanoparticle (tag).

In contrast to a cell labeled with antibody-magnetic particle conjugates, which can have on the order of a few (micron sized) to tens of thousands for nanosized particles [65], when a RBC, or cancer cell, is intrinsically magnetic, the element responsible for this magnetic moment (generally iron or magnesium) does not saturate in the fields that are typically used. Consequently, the magnetic force operating on a cell can be written as:

(3) $$F_{\text{mag}}=\frac{\Delta \chi V(B•\nabla )B}{\mu_{0}}=\Delta \chi VS_{m}$$

where Δχ is the difference in volumetric magnetic susceptibility of the suspending fluid and the cell, and V is the volume of the cell. The vector $S_{m}$, the magnetic force field strength, inspired by an analogy to electrophoresis, is defined as:

(4) $$S_{m}=\frac{(B•\nabla )B}{\mu_{0}}=\frac{\nabla B^{2}}{2\mu_{0}}$$

The vector $S_{m}$ provides a convenient way to compare various magnetic devices. In particular, the magnetic field gradient product, is defined by [66]:

(5) $$|(B•\nabla )B|=\sqrt{{\left(B_{x}\frac{\partial B_{x}}{\partial x}+B_{y}\frac{\partial B_{x}}{\partial y}+B_{z}\frac{\partial B_{x}}{\partial z}\right)}^{2}+{\left(B_{x}\frac{\partial B_{y}}{\partial x}+B_{y}\frac{\partial B_{y}}{\partial y}+B_{z}\frac{\partial B_{y}}{\partial z}\right)}^{2}+{\left(B_{x}\frac{\partial B_{z}}{\partial x}++B_{y}\frac{\partial B_{z}}{\partial y}+B_{z}\frac{\partial B_{z}}{\partial y}\right)}^{2}}$$

Eqs. (1–3) indicate how “magnetic” a cell is either intrinsically or labeled with magnetic particles, and Eqs. (4,5) indicate the magnitude of the magnetic gradient product that represents the separator strength, which is also needed to calculate the force acting on the cell and directly affects the cell separation process. The complexity of quantifying the magnitude of $S_{m}$ (Eq. 5) in HGMS devices, and thereby, the magnetic force operating on a single cell (Eq. 1) is high.

### 2.2. Models of magnetic filtration

Beyond the separation of biological cells, significant applications of HGMS for removing magnetic particles from liquids and gases have existed since the 1960 s. These processes are commonly called “magnetic filtration” and a number of theoretical models have been developed to describe the process and predict its performance [45].

One such model was developed by Abbasov and co-workers [44,46] which has similarity to a basic molecular balance approach used for chromatography. The non-linear partial-differential equations consist of a continuity equation:

(6) $$\frac{\partial \sigma (x,t)}{\partial t}+\frac{1}{\varepsilon_{0}}\frac{\partial \sigma (x,t)}{\partial t}+\frac{\nu }{\varepsilon_{0}}\frac{\partial c(x,t)}{\partial x}=0;c(0,t)=c_{0}$$

And the rate equation:

(7) $$\frac{\partial \sigma (x,t)}{\partial t}=\beta v\left[1-\frac{\alpha }{\beta u}\right]c(x,t)$$

(8) $$\sigma (\text{x},0)=0;\;\text{u}=\nu /\varepsilon_{0}$$

In this model, c(x,t) and σ(x,t) are the concentrations of the free and captured particles, respectively, α and β are the detachment and accumulation coefficients, ν is the filtration velocity of the suspension, and ε is the porosity.

Using these relationships, one can solve for c(x,t) using Laplace transforms to obtain:

(9) $$\frac{c\left(x,t_{1}\right)}{c_{0}}=e^{\left(\alpha t_{1}-\beta x\right)};t_{1}=t-t_{0}$$

Where the following initial condition is used:

(10) $$c\left(x,t_{0}\right)=c_{0}e^{-\beta x}$$

Using this solution, the magnetic packed bed performance (capture efficiency), ψ, is further defined as:

(11) $$\psi =\left(1-exp\left[\alpha \left(t-t_{0}\right)-\beta x\right]\right)$$

Abbasov (2001) [44] reported that there exists a “specific time” (for any specific magnetic filtration device) of operation prior to which all magnetic material is retained by magnetic filtration. Subsequent to this “specific time”, $t_{0}$, the value ψ declines following the functional form of Eq. (11).

$t_{0}$ can be further determined from the accumulation mechanism of the particles/cells in the spaces between the packing material [44,45]:

(12) $$t_{0}=\frac{2\rho_{\text{packing}}}{5\rho }\frac{\sigma_{s}}{\lambda \beta uc_{0}}=k_{2}\frac{1}{\beta^{\prime }c_{0}}$$

where $\rho_{\text{packing}}$ is the density of the packed material, $\sigma_{s}$ is the saturation value of the specific deposit, and u is the flow velocity (interstitial velocity. As we will present below, $k_{2}$ and $\beta^{\prime }$ are lumped variables used for experimental data fitting.

The detachment coefficient, α, can be obtained from the equilibrium equations of forces and moments acting on the particles held in the active area:

(13) $$\alpha =\frac{\beta vc_{0}}{\sigma_{s}\gamma }\frac{\rho }{\rho_{\text{packing}}}=\frac{0.4}{\gamma \lambda }\frac{1}{t_{0}}=k_{1}\frac{1}{t_{0}}$$

The accumulation coefficient, β, is defined by:

(14) $$\beta =-2.6\times 10^{-2}\left(\frac{v_{m}}{v}\right)\left(\frac{1}{d}\right)=-1.4\times 10^{-2}{\left(\frac{P_{m}}{\Delta P}\right)}^{0.6}{\left(\frac{1-\varepsilon }{\varepsilon^{3}}\right)}^{0.6}\left(\frac{1}{d}\right)$$

where, v is the bulk fluid velocity through the bed, d is diameter of the spherical packing, $P_{m}$ is the magnetic pressure, ΔP is the pressure drop in the packed column, and ε is the bed porosity.

The “magnetic velocity”, $v_{m}$, and magnetic pressure, $P_{m}$ are further defined by:

(15) $$v_{m}=\frac{3k\mu_{0}\mu^{1.38}H^{2}(1-\phi )\delta^{2}}{d\eta }$$

(16) $$P_{m}=\frac{k\mu_{0}\mu^{1.38}H^{2}(1-\phi {)}^{\delta }}{d}L_{d}$$

Where k is the magnetic susceptibility of the particle to be separated, $\mu_{0}$ magnetic permeability of free space (H/m), μ is the relative magnetic permeability of the filter packing, H is magnetic field intensity (A/m), ϕ is the accumulated particle volumetric fraction, and $L_{d}$ is the length of the magnetic filter.

While complex, Eq. (11) is fundamentally, a four-parameter model; accumulation term, β, the detachment term, α, the length of the column, x, and $t_{0}$, the time, prior to which, all magnetic particles are retained within the column. Further, Eq. (10), using the definition of these four parameters, such as β and α, can guide us in attempting to predict the effect that various adjustable parameters can have on the magnetic filtration performance. For example, Eqs. (10, 14 and 15) suggest that increasing the applied magnetic field intensity can increase the capture efficiency, ψ, to the square of the value of H.

## 3. Methods

### 3.1. Sample preparation

Whole blood was obtained from healthy individuals following protocols approved by the Institutional Review Board (IRB) at The Ohio State University (protocol number #38334). Approximately 8 mL of blood was drawn and collected into a 10 mL collection tube containing EDTA anticoagulant. The RBCs from the whole blood were washed three times using phosphate buffered saline (PBS) using centrifugation at 1300 × g for 5 mins. After washing, RBCs were divided into two aliquots (oxygenated RBCs [oxyHb-RBCs] and methemoglobin RBCs [metHb-RBCs]) for further analysis. OxyHb-RBCs were prepared by leaving the RBCs to equilibrate under ambient atmospheric conditions for 10 mins. MetHb-RBCs were prepared by treating oxyHb-RBCs with sodium nitrite following a protocol published in the literature [66].

### 3.2. Ferromagnetic packed column and applied magnetic fields

**Fig. 1.**
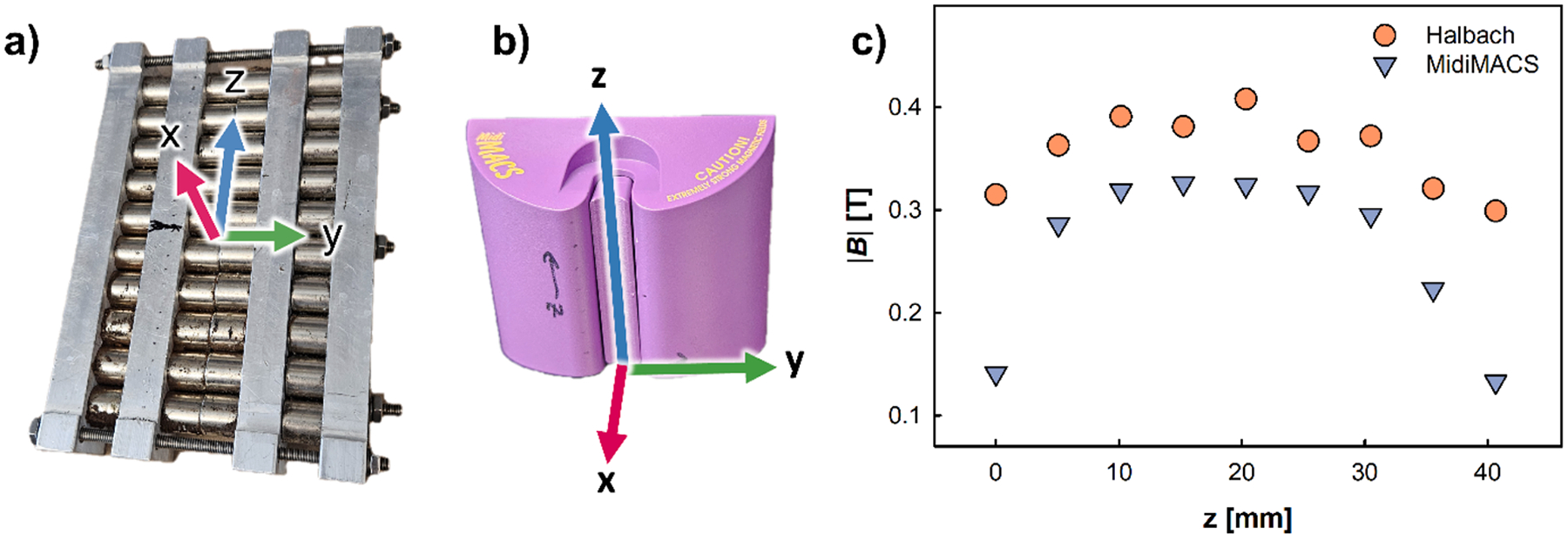
3-D schematic of the a) Halbach magnet, and b) MidiMACS magnet used in this study (MidiMACS^™^). c) Plot of |B| as a function of the z-direction along which the LS column was placed for the experiments conducted in this study.

**Fig. 2.**
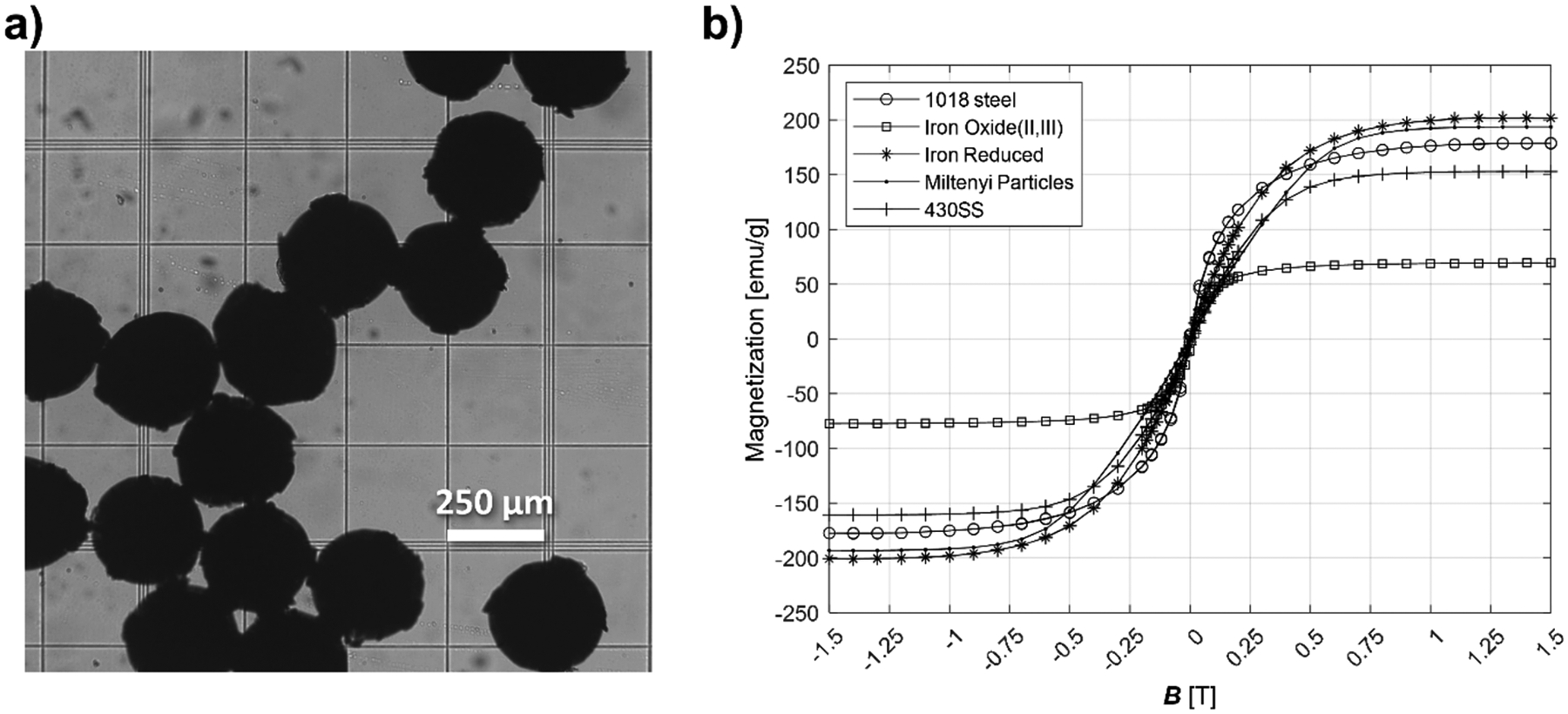
A) image of the packing beads contained in a miltenyi ls column. b) experimentally measured magnetization curve for 1018 steel, iron oxides ii and iii, reduced iron, miltenyi packing beads, and 430 stainless steel. measurements were made on a lakeshore 7400 series vsm.

For performing the magnetic cell separation, Miltenyi MACS^™^ LS columns (Cat# 130-042-401, Miltenyi Biotec) were used. The magnetic field was generated by two different magnetic assemblies: 1) a Halbach array; and 2) a Miltenyi MidiMACS^™^ separator (Cat# 130-042-302, Miltenyi Biotech). The Halbach array is discussed in our previous study [67]. Fig. 1a and 1b provide 3-D drawings of the Halbach array and the MidiMACS^™^ magnets, respectively. Fig. 1c presents the measured external magnetic field of the same location as where the center of the column is placed. The column was positioned along the z axis of Fig. 1a and b, and the direction of flow is opposite from z-axis. The region of the LS column that contains ferromagnetic packing has approximate outer diameter of 0.8 cm and a height of 4.2 cm. The wall thickness of the column is approximately 1 mm, which yields a total packing volume of 1.1 cm^3^. Miltenyi Biotech reports that the void volume is 400 μL, thus the void fraction is 0.35 (35 %). In order to characterize the matrix, we removed the packing within the LS column, imaged it, and measured the magnetic characteristics of the material using a Lakeshore 7400 series vibrating-sample magnetometer (VSM). Fig. 2a provides an image of the particles in the packing, and Fig. 2b presents the measured magnetization as a function of the magnetic field for these particles, along with 1018 steel, and iron oxide (II and III).

In the cell sorting experiments, the LS column was attached to each magnetic field source and a collection tube was placed under the LS column. 2 mL PBS was used to rinse the column, and then a 1 mL sample containing RBCs with known concentration was fed into the column. Once all the fluid eluted through the column, 12 mL PBS was used to wash the column. The cells collected in the collection tube consisted of “non-captured” cells. After performing washing steps to remove “non-captured” cells, the LS column was removed from the magnet and placed in a new collection tube. “Captured” cells were obtained by immediately flushing the column with 4 mL of PBS three times using a plunger.

### 3.3. Coulter counter analysis

The cell concentration of each sample (“non-captured” and “captured”) was measured by using an automated cell counter, B23005 Multisizer 4e Coulter Counter (CC, Beckman Coulter, CA). By carefully using a pipette to measure the volume of samples obtained, it is possible to further calculate the total number of cells in each sample volume from the cell concentration and the measured volume.

The capture/separation efficiency of the column was defined as the ratio between the number of captured cells and the sum of captured and non-captured cells as follows:

(16) $$\text{captureefficiency}=\frac{\text{\#capturedcells}}{\text{\#capturedcells}+\text{\#noncapturedcells}}*100\%$$

### 3.4. Cell tracking Velocimetry analysis

In addition to measuring cell size and concentration with the Coulter Counter, the Cell Tracking Velocimetry (CTV) was used to characterize the magnetic behavior of the captured cells and the non-captured cells. CTV is an instrument that quantitatively characterizes the magnetic properties of individual biological entities by tracking motions under applied magnetic and gravitational fields, and consists of permanent NdFeB magnets, a microscope, a CCD camera and a microfluidic channel [39]. More specifically, the magnetophoretic and gravitational movement of cells can be recorded in the region of interest and the velocities in horizontal (magnetophoretic) and vertical (gravitational) directions can be expressed as:

(17) $$u_{m}=\frac{\left(\chi_{\text{Cell}}-\chi_{\text{Fluid}}\right)V_{\text{cell}}}{3\pi D_{\text{Cell}}\eta }S_{m}$$

(18) $$u_{s}=\frac{\left(\rho_{\text{Cell}}-\rho_{\text{Fluid}}\right)V_{\text{cell}}}{3\pi D_{\text{Cell}}\eta }g$$

where $\chi_{\text{Cell}}$ and $\chi_{\text{Fluid}}$ are the magnetic susceptibilities of the cell and the buffer, respectively; $\rho_{\text{Cell}}$ and $\rho_{\text{Fluid}}$ are the densities of the cell and the fluid, respectively; $D_{\text{Cell}}$ and $V_{\text{cell}}$ are the diameter and volume of a cell, respectively; η is the viscosity of the suspending fluid; and g is the acceleration due to gravity (9.8 m/s^2^). To normalize the magnetic velocity to the field gradient, mobility is defined as:

(19) $$m\equiv u_{m}/S_{m}$$

### 3.5. Simulations of the magnetic field and cell trajectories

While the details of the Halbach system have been reported [67], comprehensive specifics regarding the MidiMACS column holder are not available beyond the experimental findings presented in Fig. 1b. These experimental measurements indicate a reasonably uniform magnetic field distribution across the gap housing the LS column. Consequently, the simulations of the ferromagnetic packing were carried out using the Finite-Element Method (FEM) software, COMSOL Multiphysics 6.0. These simulations comprised two distinct components: the magnetic field gradient with magnetization of ferromagnetic packing and the movement of metHb-RBCs within the ferromagnetic packing.

**Fig. 3.**
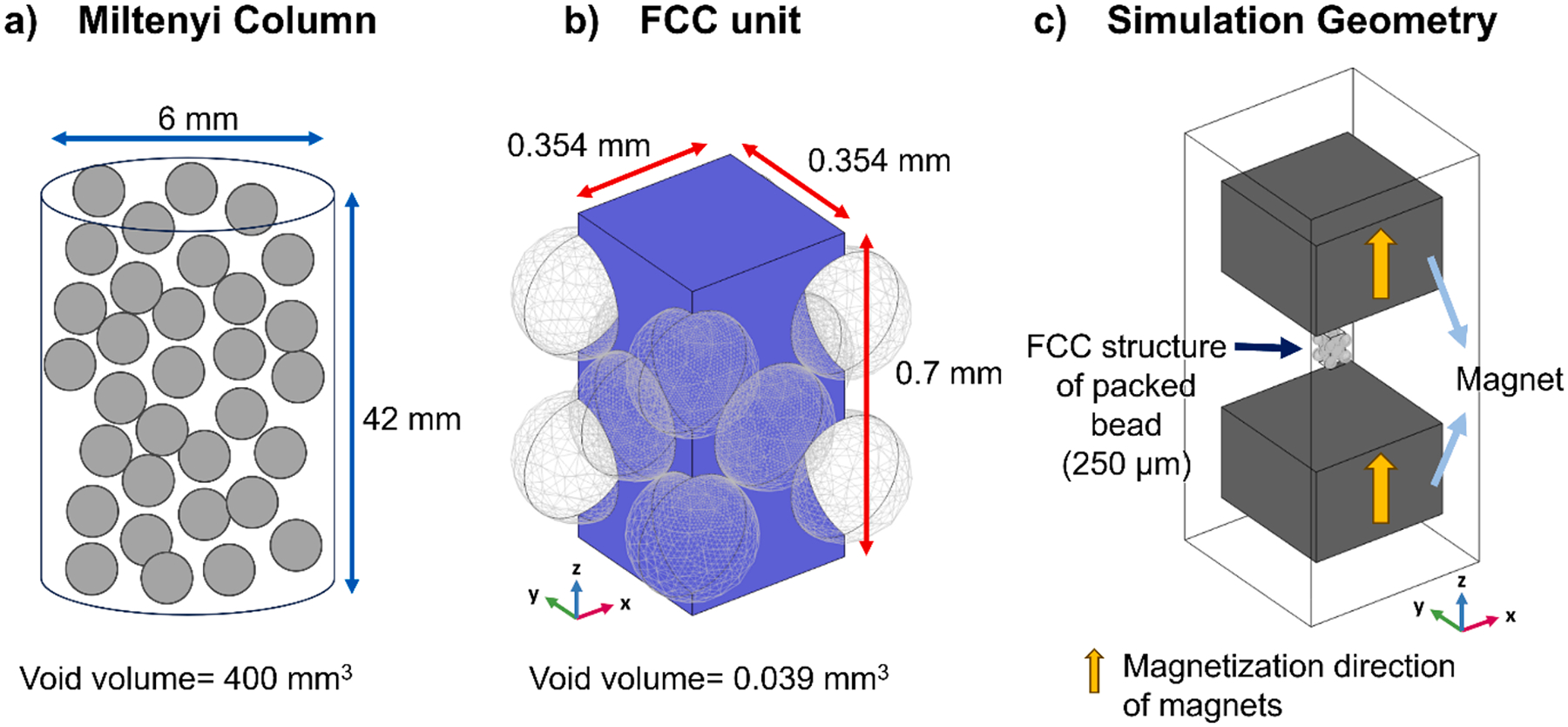
Schematic figures of a) Miltenyi packed column and b) FCC unit, and c) location of the FCC structure of beads in between two block magnets.

The magnetic field was first investigated using the “Magnetic Fields, No Currents (mfnc)” physics module within the AC/DC module, to model the magnetic flux density (B) and the magnetic field intensity (H). To construct the internal geometry in the ferromagnetic packing, we configured the face-centered cubic (FCC) unit in a manner designed to replicate the Miltenyi column, as visually represented in Fig. 3a. A total of 14 magnetic sphere beads, each with a diameter of 0.25 mm, arranged in an FCC structure with a side length of 0.354 mm, as depicted in Fig. 3b. The externally simulated magnetic field was generated by two blocks, acting as permanent magnets, each measuring 3 mm × 3mm × 2 mm, positioned at ± 2.5 mm along the z-axis relative to the origin as shown in Fig. 3c. These permanent magnetic blocks serve to create the external magnetic field, with custom material properties characterized by a recoil permeability of 1.05 and remanent flux density norms of 1.66, 5, 6.66, 8.3, 16.6, and 33.3, yielding magnetic fields of 0.1, 0.3, 0.4, 0.5, and 1.0 T at the origin point, respectively. The 14 spherical ferromagnetic beads, arranged in the FCC structure, were positioned at the coordinate origin point and exhibited the physical properties of low carbon steel pure iron sourced from the COMSOL Multiphysics material library. The magnetization model for these beads was set as “B-H curve” to account for the nonlinear magnetic characteristics of pure iron, which are already contained in the material properties. To enhance the resolution of the field of view at the center of the FCC structure, a block with dimensions of 0.177 mm was incorporated at the origin point as the central position. Additionally, the mesh element size of this block was generated using mesh operator in COMSOL with the setting of calibrated for “Fluid dynamics” and predefined “Extremely fine” option, ensuring a high-resolution analysis.

To investigate the behavior of metHb-RBCs within the ferromagnetic packing system, we utilized the “Laminar Flow” and “Particle Tracing for Fluid Flow” modules. Prior to conducting the particle tracing, the fluid flow around the FCC unit was calculated using a stationary solver, with water as the fluid material. To simulate the practical fluid flow from the inlet to the outlet of the Miltenyi column, a cuboid with dimensions of 0.354 mm × 0.354 mm × 0.7 mm was introduced. The inlet boundary condition was set to “Fully developed flow” with the flow rate varied within a range of 10^−5^–10^−2^ mL/min, and the outlet boundary condition was set to “Pressure” with a static pressure of 0 Pa.

For the particle tracing, the properties of metHb-RBCs in the simulations were derived from the existing literature [28,68], with a density value of 1050 kg/m^3^, diameter of 5 μm, and a magnetic susceptibility of 0.301 × 10^−6^. The magnetic susceptibility of water was −9 × 10^−6^. In each simulation, 100 particles were released at 0 sec from random initial position at the inlet of the cuboid. This number was chosen to facilitate easy conversion of results into percentages, allowing for straightforward interpretation and comparison of the particle behavior within the system. The model comprehensively integrated magnetophoretic, gravitational, and drag forces, based on magnetic field and flow velocity values from previous calculations. Additionally, a “freeze wall” boundary condition was applied to halt cell movement upon collision with a wall. Given this setting, cells that touched the box wall would also stop at that position, so a particle counter was applied to the surface of the FCC beads to separately count cells captured by the FCC. A time-dependent solver was deployed to update cell positions and velocities at each time step in response to the forces acting upon them. The trajectories of the particles were systematically monitored over time, enabling an evaluation of the cell behavior within the magnetic field. The simulation was conducted over duration ranging from 1 to 60 s, depending on the flow rate, to ensure that not captured cells reached the outlet. Since the particles were assumed not to interfere with the fluid flow, and to expedite the calculations, a one-way coupling approach was used for fluid-particle interactions in the particle tracing simulations.

## 4. Results

### 4.1. Magnetic separator field characterization simulations

As presented previously, and mathematically represented by Eqs. (1–4), the magnetic force acting upon a RBC, or a cancer cell containing iron, is proportional to the magnetic force field strength ($S_{m}$). Thus, increase of B will result in an increase in the magnetic force operating on a cell by $B^{2}$, all other factors being held constant. As presented in Fig. 2b, the beads in the magnetic column saturate at values of B significantly higher than beads consisting of ${\text{Fe}}_{\text{x}}{\text{O}}_{\text{y}}$ which are typical used in “SPIONS”, such as in the magnetic nanoparticle conjugation with antibodies. Further inspection of Fig. 2b suggests that the packing beads in the Miltenyi MACS^™^ LS columns begin to saturate above 0.5 T; however, the effective saturation does not occur till B is on the order of 1 T, if not higher.

**Fig. 4.**
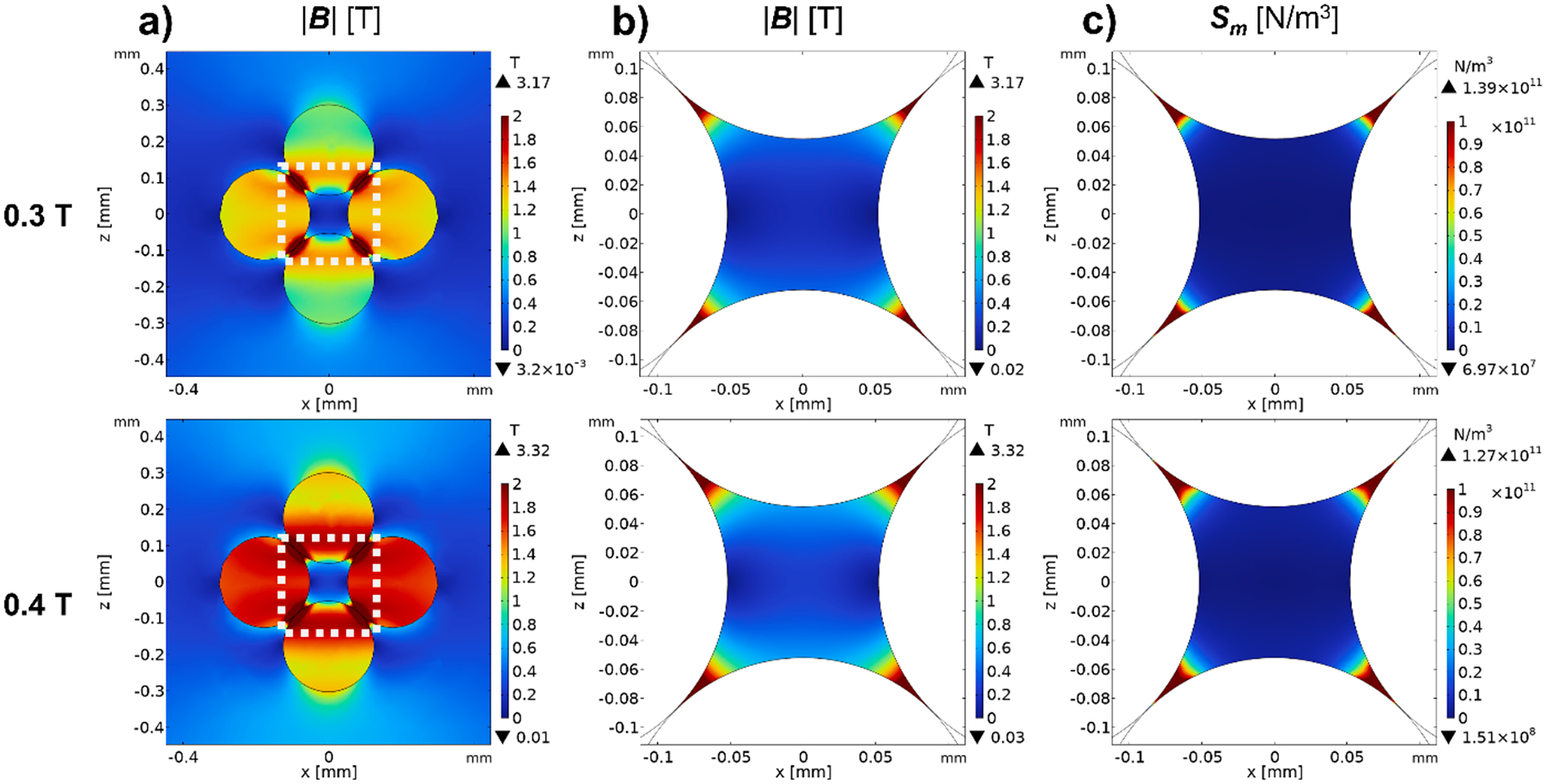
A) representative 2-d slice of |B| in the open zone contained within the FCC packing. The top slice corresponds to the maximum external imposed magnetic field measured using a 0.3 T external field, and the lower plot corresponds to the maximum external imposed magnetic field measured using a 0.4 T external field. b) Magnified slices of open zone between the spheres closely contacting each other. This area is corresponding with the white box in a). c) Presentation of similar slices (along the Z axis) for values of $S_{m}$, for the same two magnet systems.

Fig. 4 presents COMSOL simulation heat maps of magnetic field resulting from the placement of a FCC structure (Fig. 3c) in a 0.3 T and 0.4 T external field, such as created by the MidiMACS and Halbach magnet (Fig. 1c). Fig. 4a and b are representative two-dimensional cross-sectional slices, x and z axis (see Fig. 3a for coordinate directions) that displays the magnitude of the magnetic field intensity, denoted as |B|, for all values of z within the open region enclosed by the FCC packing. The upper slice corresponds 0.3 T, the lower slice to 0.4 T. Fig. 4c are corresponding cross-sectional slices, with only the values of the magnetic force field strength ($S_{m}$), at the same corresponding x-z slice. Qualitatively, these results are consistent with the generally accepted concept that the highest values of B, and the corresponding gradients are associated with the areas close to where two spheres approach each other.

**Fig. 5.**
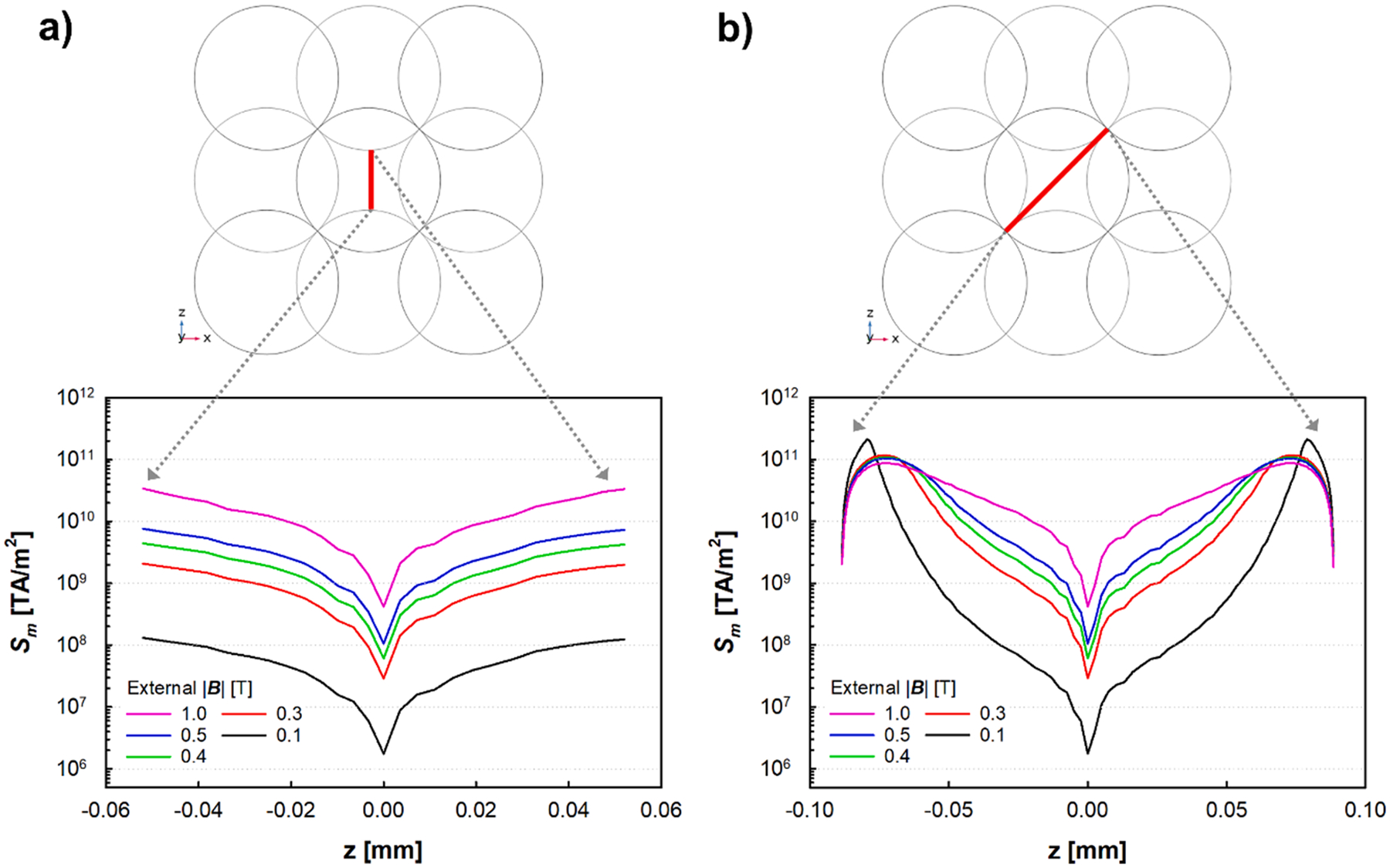
2-D cut of the simulated FCC structure showing in a) a vertical line, shown in red, while in b) this line is presented in diagonal, being both lines traversing the open zone between the beads that form the packing material of the column. Complimentary to providing |B| values, lower graphs provide simulated $S_{m}$ values as a function of the length of the corresponding red line, for a range of externally applied |B| values.

To provide further, quantitative representation of the computer simulation results, a two-dimensional cross-sectional view of the FCC unit is provided in Fig. 5a and 5b. In Fig. 5a, a vertical red line is depicted, while in Fig. 5b, this line takes on a diagonal orientation, with both lines traversing the open space between the spherical beads. The simulated $S_{m}$ values are presented across these two red lines for a range of externally imposed values of |B|, from 0.1 to 1.0 T.

### 4.2. Experimental data

The COMSOL simulations demonstrate that despite a moderate increase in the applied magnetic field of approximately 0.3–0.4 T (Fig. 1c), an almost 2-fold increase in $S_{m}$, and correspondingly magnetic force on RBC, results from the use of the Halbach magnet.

**Fig. 6.**
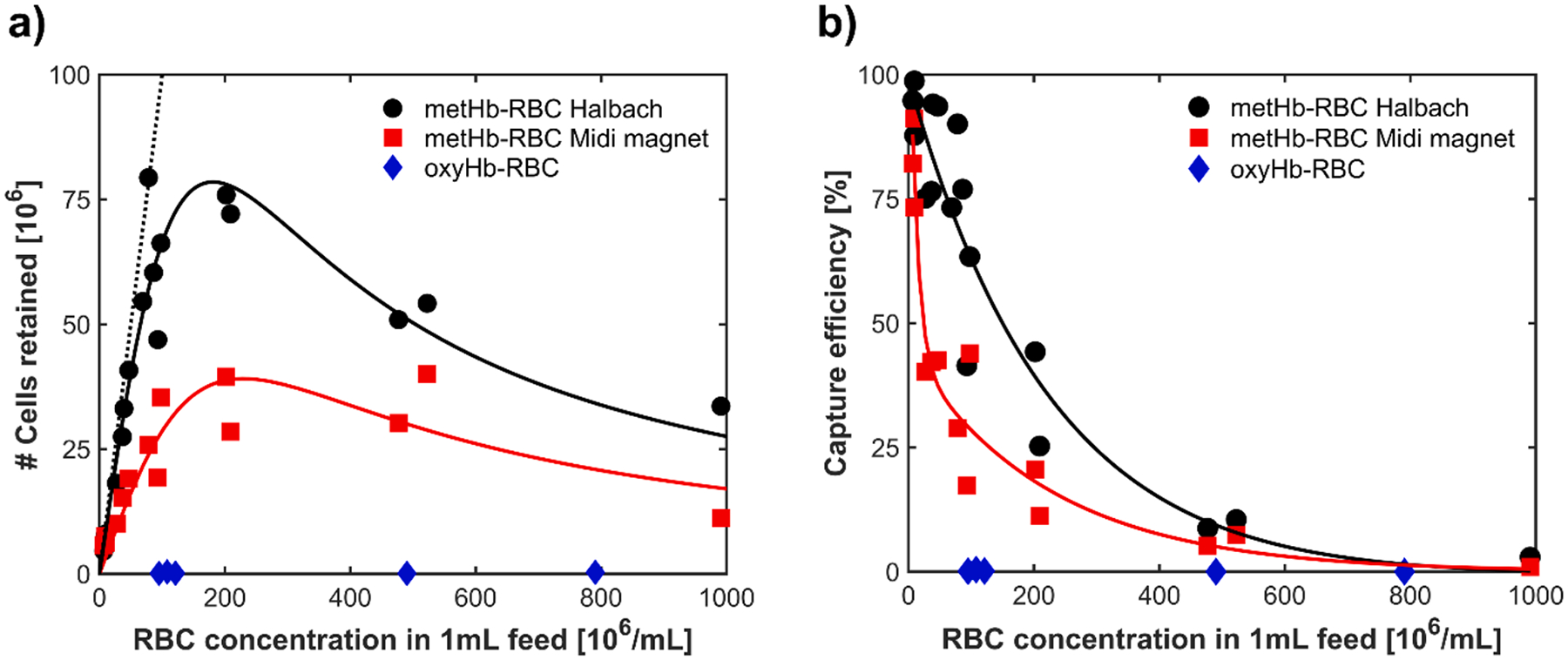
A) number of methb-rbcs retained by the ls packed column separator using the two magnet systems discussed above. the data points correspond to the total number of cells retained by the column as a function of the feed concentration. as a control, data for oxyhb-rbcs (diamagnetic) separations are also presented. b) capture efficiency of the packed column using the two separate magnet systems.

Given these observations, we initially attempted to separate met-RBCs in the commercial Miltenyi Columns with the standard Miltenyi magnetic, MidiMACS^™^, and our higher power Halbach magnet. Further, following the company protocol, we introduced the cell suspension in the vertically oriented magnetic column and let the cell suspension flow through the column as a result of gravity. To further facilitate operation and analysis, a range of cell concentrations, ranging from 1.8 to 2.3 × 10^8^ cells/mL was used, and for all experiments, the column was previously “wetted” with just the buffer solution; prior to the buffer level approaching the packing, (i.e. fluid was flowing through the column), 1 mL of the specific cell concentration was added. When this cell suspension also approached the packing, 12 mL of buffer was added as a “chase”. The volume, and concentration of cells of the fluid that exited the column (with the column still in the magnetic field) was determined. Further, the column was then removed from the magnetic field, the column further flushed with buffer and the collected fluid was measured for cell concentration and volume. From these collected cell suspensions, the performance of the experiments was determined, and presented in Fig. 6.

From this Figure, a number of interesting, and perplexing results were obtained. First, for the higher field Halbach system, for cell concentrations below 5 × 10^7^ cells/ml, and 1 mL of cell suspension, a high percentage of the cells were retained, i.e. the dotted black line indicates a slope of 0.87. Secondly, the cells retained using the MidiMACS magnetic had a performance that was noticeably less, a slope of 0.34. This is consistent with the higher values of $S_{m}$ from the COMSOL simulations.

Furthermore, as depicted in Fig. 6b, the capture efficiency is presented. It is evident that an increase in the feed cell concentration results in a large decline in capture efficiency with either magnet, ultimately reaching zero when the feed cell concentration approaches 1 × 10^9^ cells/mL. In accordance with the trend observed in Fig. 6a, the use of the Halbach system provided a notably higher capture efficiency compared to that of the Midi magnet, exhibiting nearly twice the increase when the feed concentration is 1 × 10^8^ cells/mL. This finding underscores the enhanced performance of the Halbach system in the metHb-RBC capture, particularly at relatively lower feed concentrations.

What is perplexing with these results is the drop in the number of cells retained as the concentration of cells increased, Fig. 6a. This phenomenon may be related to the rouleaux effect, where RBCs form aggregates resembling stacks of coins. This arrangement is primarily due to interactions between plasma proteins and RBCs, especially under low shear flow conditions. In this stacked formation, blood viscosity increases significantly because the aggregates have a larger and more irregular shape compared to individual cells [69,70]. The higher viscosity creates greater resistance to flow, reducing cell mobility and impairing their ability to migrate and interact with capture surfaces. This can prevent cells from reaching areas where they could be magnetically captured, thus lowering capture efficiency. A further observation in these studies, and a general observation with the use of such packed columns, is that the flow rate through the system can significantly vary.

**Fig. 7.**
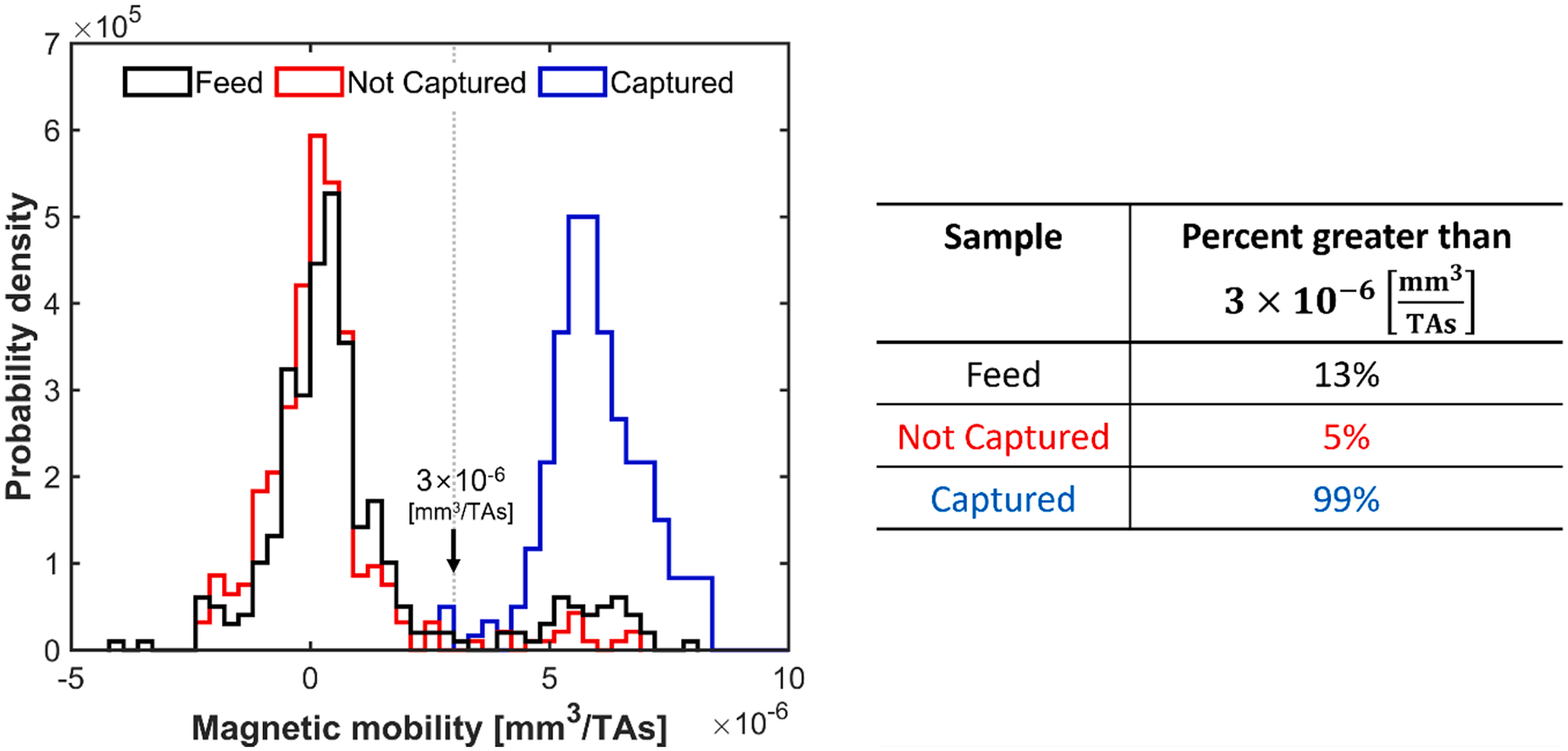
Magnetophoretic mobility histograms of feed, captured, and non-captured fractions from a Miltenyi column separation in the Halbach magnet. The feed mixture was targeted to a 9:1 ratio of oxyHb-RBCs to metHb-RBCs.

In addition to studying the relationship between cell retention and cell loading, with a relatively homogeneous population of metHb-RBCs (Fig. 6), studies were conducted with a heterogenous mixture of artificially generated oxyHb-RBCs and metHb-RBCs. Fig. 7 shows CTV measurements of the separated fractions. The feed mixture was targeted to be a 9:1 ratio of oxyHb-RBCs to metHb-RBCs. From CTV measurements, we observed that approximately 15 % of the RBCs in the feed had a magnetophoretic mobility characteristic of metHb-RBCs. As expected, the magnetophoretic mobility distribution of the captured RBCs (retained by the column when in the magnetic field) indicates a nearly pure metHb-RBC population. In contrast, the fraction that was not retained by the column had a significantly reduced population of metHb-RBCs, with only 6 % of the RBCs in this population displaying the mobility signature of metHb-RBCs.

### 4.3. Simulated and experimental effects on flow rate through the packed column

**Fig. 8.**
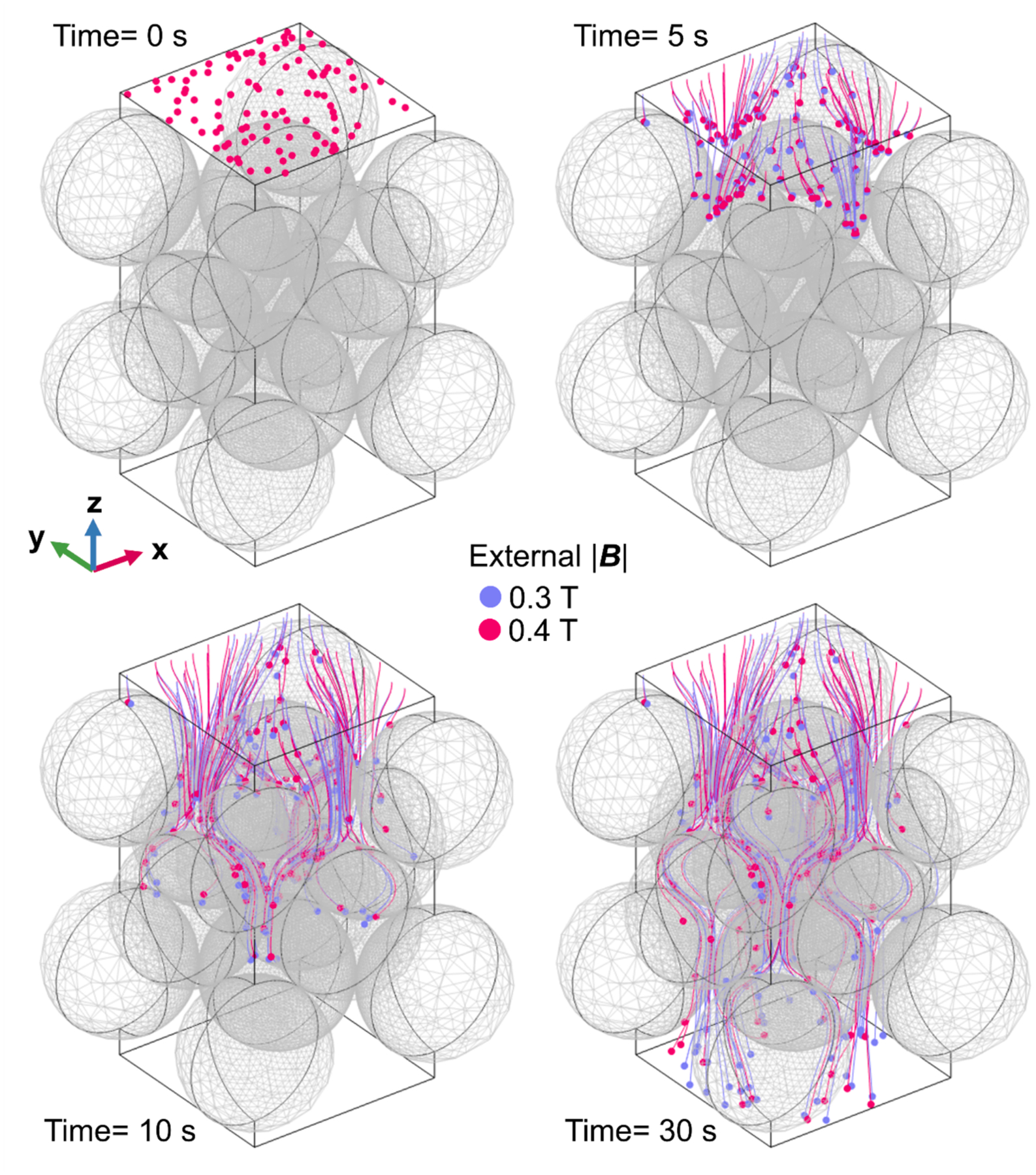
Graphical depiction of particle trajectories within an FCC unit, derived from computational simulations. The visualizations are shown at t = 0 s, t = 5 s, t = 10 s, and t = 30 s. These simulations include two different magnetic field intensities: represented in blue for 0.3 T and pink for 0.4 T.

To determine the effect of flow rate on RBC separation in the Miltenyi columns, COMSOL simulations were conducted, and guided by these simulations, actual experiments were subsequently executed. The fluid flow rates were systematically varied within the range of 10^−5^–10^−2^ mL/min across multiple trials, which correspond to 0.102–102.09 ml/min for Miltanyi column. The entire course of particle trajectories during the simulation was recorded as images, and a selected subset is presented in Fig. 8 for reference, with flow rate of 10^−4^ mL/min.

During this simulation process, particles either successfully traversed through the FCC unit or stopped on a packing surface. The location of these “stopped particles” in general corresponded to the areas of high values of $S_{m}$. (see Figs. 4 and 5). Furthermore, at an elevated magnetic field strength of 0.4 T (illustrated by the pink data points), a greater number of particles were observed to be entrapped within the FCC unit, as compared to the conditions prevailing at 0.3 T (denoted by the blue data points).

**Fig. 9.**
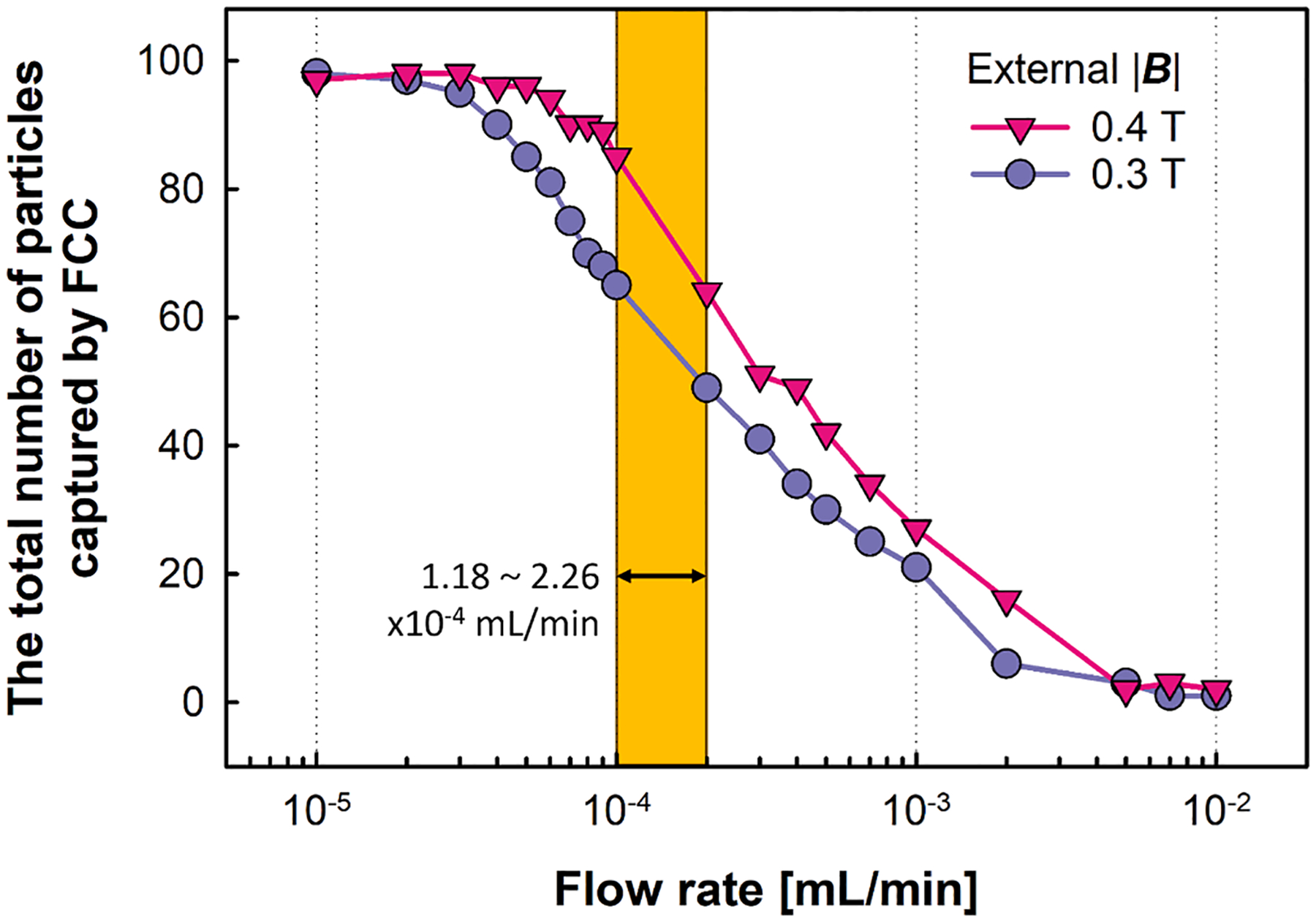
Total number of particles captured by the FCC unit when applying a 0.3 and 0.4 T magnetic field as a function of flowrate, estimated numerically. The highlighted region represents the range of flowrates under gravity (1–2 ml/min in Miltenyi column) that were observed experimentally.

To quantitatively assess the impact of the magnetic field intensity and flow rate on the efficiency of the separation process, the number of particles captured within the column was plotted as a function of flow rate, Fig. 9. These conditions include two magnetic field strengths (0.3 and 0.4 T) in conjunction with a range of flow rates ranging from 10^−5^ to 10^−2^ mL/min. A clear effect of flow rate and magnetic field intensity effect particle capture results. Additionally, when the flow rate is increased from 10^−4^ to 10^−3^ mL/min, there is a substantial reduction in the number of particles entrapped within the FCC unit, amounting to a nearly fourfold decrease. These simulated flow rates within the FCC unit, 1.18–2.06 × 10^−4^ mL/min, corresponds to the experimental bulk flow rate range of 1.2–2.1 mL/min [71], if one assumes a simple scale up to the Miltenyi columns used in this study. Additionally, it was noted that at a flow rate above 10^−2^ mL/min, all particles travel through the FCC unit without experiencing any entrapment.

COMSOL numerical simulations results were analyzed in JMP to determine the effects of the process variables (flowrate, external field strength, and particle size). Table 2 outlines the domains of variables investigated in COMSOL with their associated log worth on influencing filtration efficiency. Results of the multifactorial COMSOL investigation yielded significant effects from all process variables. However, particle size and flowrate were found to be of nearly 33 % more log worth than the external field strength. These results indicate that flow rate control is the most significant process variable to control the separation efficiency under these ranges of variables.

**Fig. 10.**
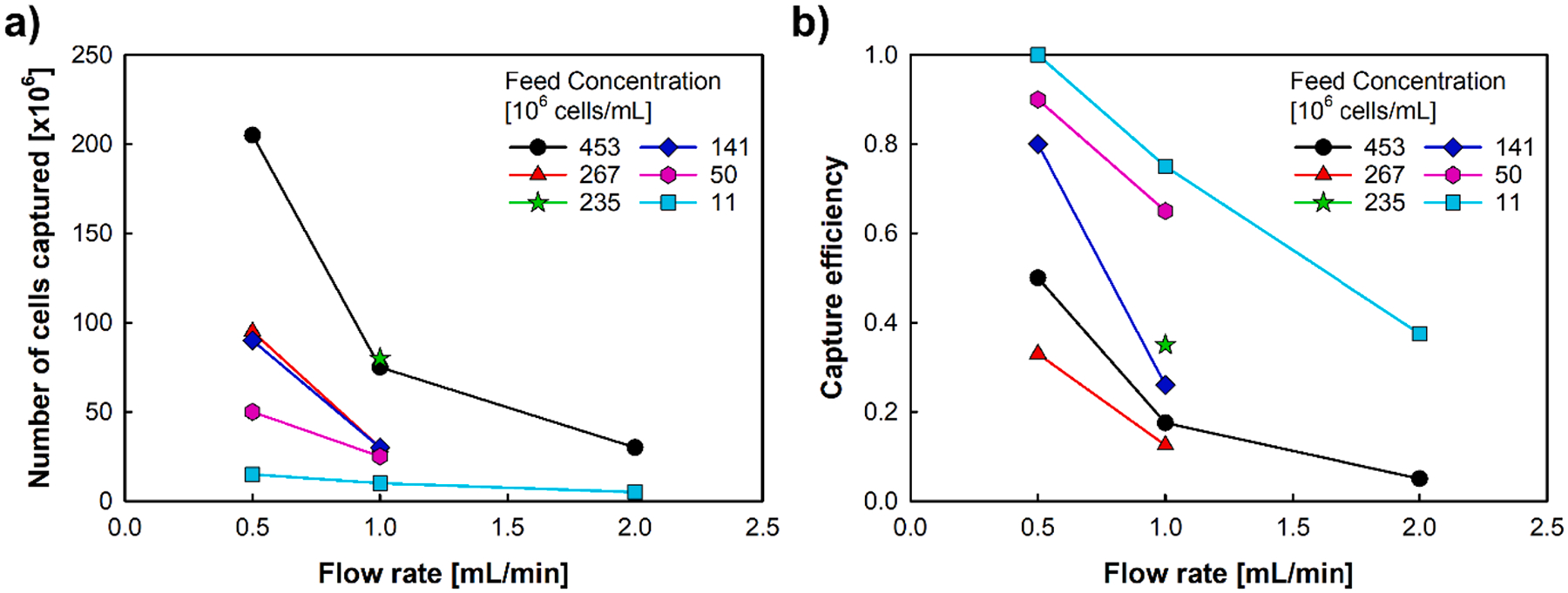
The effect of flowrate and feed concentration on the separation performance in the 0.4 T Halbach magnet: total number of cells retained (left) and capture efficiency (right).

To test these COMSOL predictions, the flow rate of the cell suspension through the magnetic column was controlled (as opposed purely to gravity fed), by a syringe pump connected to the outlet at the bottom of the Miltenyi column. Flow rates of 0.5, 1, and 1.5 mL/min were used with a RBC feed concentrations ranging from 1.1 × 10^7^ to 4.5 × 10^8^. (Fig. 10).

Consistent with the results presented in Fig. 10 and previously, and the ANOVA analysis of the COMSOL simulations, both the magnitude of |B|, and the flow rate have a strong impact on the performance of Miltenyi column. Unfortunately, as highlighted above, the use of higher concentration of particles in the COMSOL simulations resulted in the simulations not converging; however, it is significant to note that the simulations that did converge correspond to the experimental data for a feed concentration of 11 × 10^6^ cells/mL. Also note, that for all of the concentrations tested, the total number of cells captured decreased with increasing flow rate.

### 4.4. Abbasov modeling

The Abbasov model, Eqs. (6–13), includes several parameters that can be tested using commercially available columns and commercial magnets or, in this current study, more powerful magnet systems. While complex, this model is fundamentally, a four-parameter model; 1) an accumulation term, β, 2) the detachment term, α, 3) the length of the column, x, 4) and $t_{0}$, the time, prior to which, all magnetic particles are retained within the column. For the purpose of experimental modeling, Equation 11 was simplified by substitution of Eq. (12) and Eq. (13), resulting in the below equation.

(20) $$\psi =\left(1-exp\left[\alpha \left(t-t_{0}\right)-\beta x\right]\right)=1-exp\left[k_{1}\frac{t-k_{2}\frac{1}{\beta^{\prime }c_{0}}}{k_{2}\frac{1}{\beta^{\prime }c_{0}}}-\frac{\beta^{\prime }x}{v}\right]$$

Eq. (20) was used to model the effects of flow rate and feed concentration in the commercial, Miltenyi column.

In general, experimental studies of higher feed concentrations led to a higher total number of cells retained; however, this resulted in a reduced capture efficiency. Abbasov’s magnetic filtration model (Equation 20) was subsequently fit to these data (n = 13). While a range of cell concentrations and flow rates were tested, 1.1 × 10^7^–4.5 × 10^8^ cells/mL, and 0.5–2 mL/min, only one column length was tested, the length of the Miltenyi column. The fitting parameters are shown in Table 3. JMP was used to perform the nonlinear fit based on leastsquares regression. The total root mean square error, which quantifies the absolute magnitude of the prediction error, was 0.175. The experimentally determined model parameters were then used to extrapolate the performance, ψ, of the RBC magnetic filtration as a function of filter length, filtration duration, feed concentration, and flow rate.

**Fig. 11.**
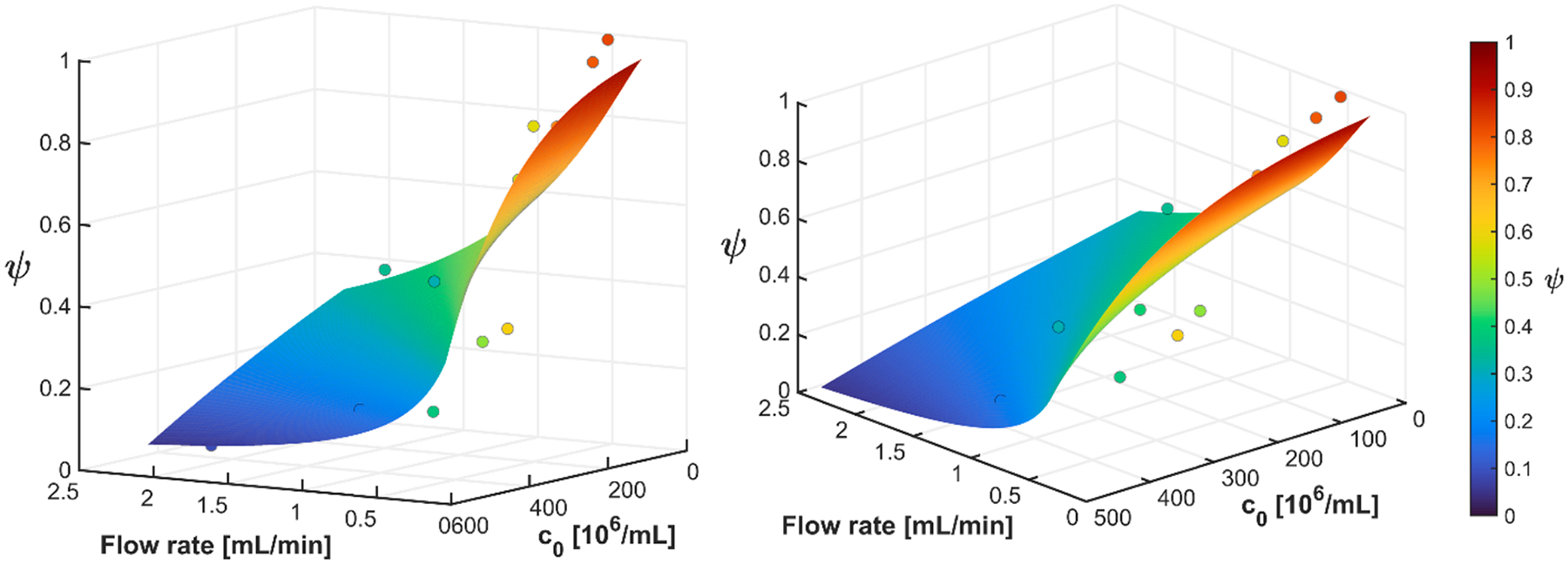
3D surface plot of magnetic packed bed performance calculated using the Abbasov magnetic filtration model with the parameters from Table 2. The individual points are the actual data used to calibrate the model.

As an alternative to presenting the fit of the model to the data using a root mean square error value, Fig. 11 presents a surface for the solution to this model, and the actual data used to fit the model are the individual datum points. In this 3-D surface plot, the magnetic packed bed performance (capture efficiency), ψ, is plotted (z-axis) as a function of both the flow rate, and feed concentration. Two slightly different perspectives are presented to assist in visualizing the data and surface.

**Fig. 12.**
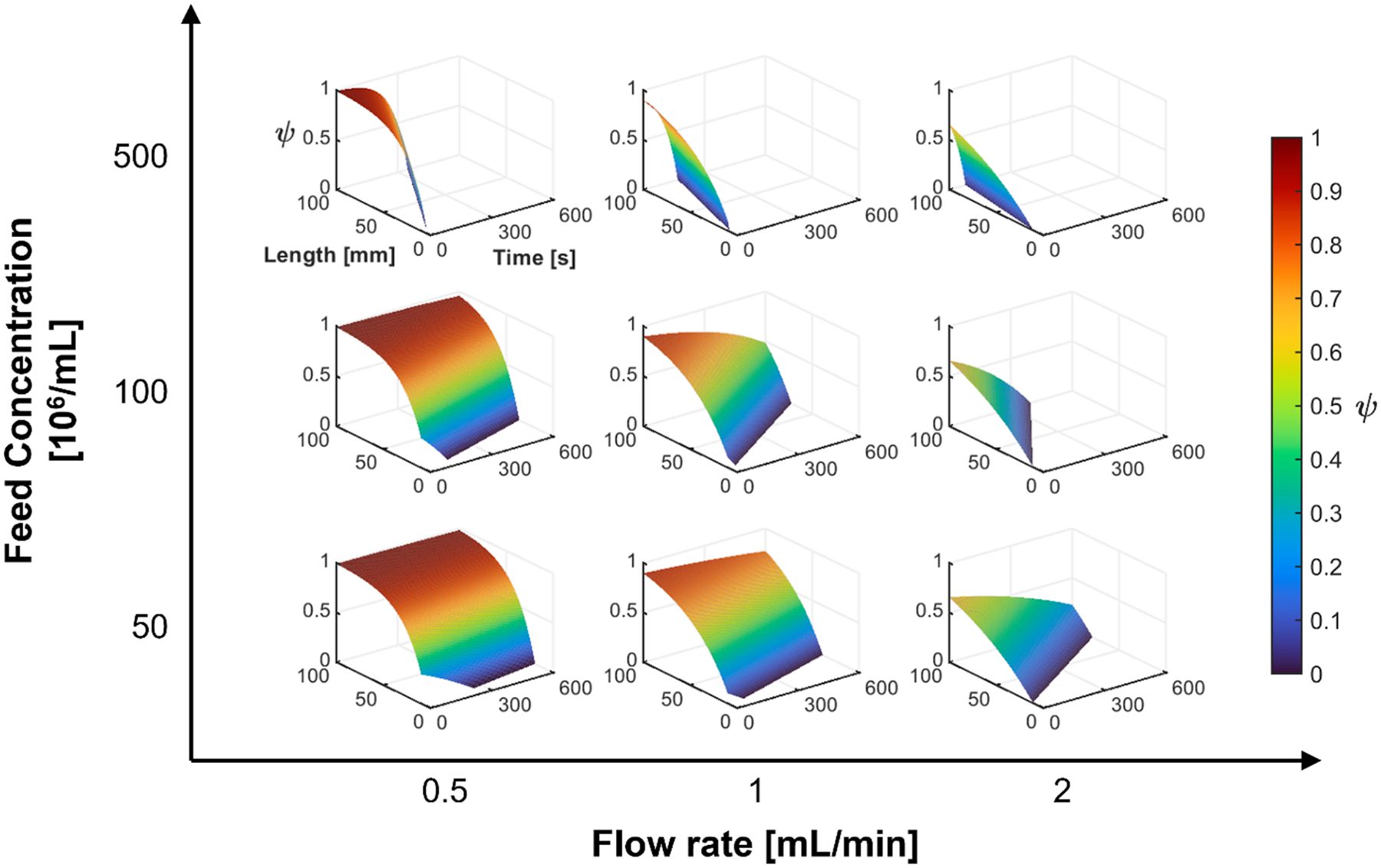
Magnetic packed bed capture efficiency predicted by the Abbasov magnetic filtration model using parameter extrapolated from RBC separations in a Miltenyi column. Model was constructed based on experimental model fit parameters and shown as a function length of column and time, for several flowrates and feed concentrations.

Fig. 12 model predictions over a wider range, and number of variables. Specifically, again the magnetic packed bed performance (capture efficiency), ψ, but this time as a function of the column length and time of operation of the filtration process. Since this only presents two of the four groups of variables in the model, a total of nine plots are presented, using three different feed concentrations, and 3 different flow rates. While it is always risky (with respect to accuracy) to extrapolate models beyond the data used to generate the model (for example, in this case, only data for a column length of 42 mm was used), we suggest that these plots can guide us in future studies.

On the other hand, the magnetic filtration performance reduces exponentially with flowrate and concentration as shown in Fig. 5. Based on a multi parameter model, process optimization could be performed depending on the application. Moreover, RBC separation by magnetic filtration demonstrates itself to scalability. Principally, the variables of concern are RBC concentration in the feed and the flowrate of the feed. Filtration length and filtration duration can be scaled depending on the size of the system and on the application. One potential application of magnetic filtration could be in the separation of RBCs from peripheral blood. Peripheral blood contains approximately 5 × 10^9^ RBCs/mL. Through a choice of flowrate and filter length, it could be conceivable to use magnetic filtration for the clearance of RBCs from whole blood.

## 5. Conclusions

In this work, we began to investigate the factors controlling magnetic filtration (retention) of RBCs using a commercially available, Miltenyi LS columns. By combining Multiphysics computational fluid dynamic software, COMSOL, first principle mathematical modeling of magnetic filtration developed for industrial magnetic particle applications, and experimental data, we have begun to elucidate factors that significantly affect the effectiveness of magnetic separation of RBCs in a packed column.

As might be expected, increasing the magnetic field applied to the magnetic column increased the number of RBCs retained. Fig. 6 indicated that a twenty five percent increase in the values of B resulted in approximately a doubling of the number of RBCs retained, over a limited number of total cells retained. Given that theory suggest that the magnetic force acting on an RBC will increase with the square of B, this is not surprising. A further, not necessarily obvious observation is that the flow rate of the cells suspension through the column can have a very strong impact on the number of RBCs retained. Both the COMSOL simulations and corresponding experimental studies presented a surprising close correlation with respect to the flow rate, above which, the number of cells retained rapidly drops.

However, what was not as obvious was the observation that a maximum number of RBCs retained in a packed column can be reached, and under some operating conditions, simply using a higher cell concentration can result in a significant decrease in the total number of cells retained, Fig. 6a. While it is not completely clear at this time for the cause of this observation, inspection of the model proposed by Abbasov, Equation 20, suggests a competition between and attachment and detachment term, each of which is composed of a number of operational variables. Our limited experimental studies, significantly limited by the use of a commercial, magnetic packed column, indicates an acceptable fit of the column performance to this model. Further studies are needed to determine if this model can explain the experimental behavior presented in Fig. 6a.

High-gradient magnetic separation (HGMS) has been extensively used in fields such as mineral processing, water purification, and biotechnology. Within biotechnology, we propose that magnetic removal of red blood cells (RBCs) using MACS separators could compete with traditional removal methods, such as centrifugation and red cell lysis. This approach could also have diagnostic applications for blood disorders like malaria or sickle cell anemia.

Focusing on sickle cell anemia, patients frequently undergo routine RBC transfusions during crises, and it has been observed that the waste apheresis product from such procedures contains a mixture of normal and sickle RBCs. Research conducted in our laboratory, as well as other studies, indicates that sickle RBCs exhibit sufficiently distinct magnetic properties compared to normal RBCs. This opens the possibility of scaling up magnetically packed columns to selectively remove sickle RBCs from the discarded fractions of clinical blood cell exchange procedures.

Developing such a device for clinical applications will require significant optimization of the magnetic column separator’s design. It is important to note that Miltenyi MACS separators were not originally designed for RBC separation, as RBCs are approximately 1,000 times less magnetic than leukocytes (when magnetically labled with MACS beads). Before scaling up, optimizing the column’s magnetization to effectively separate RBCs is critical.

The preliminary studies presented in this manuscript identify potentially important variables to consider for optimization. Other significant factors, which are not extensively discussed here but are essential from first principles and insights from the Abbasov model, include the size, shape, and material composition of the column packing, as well as the column’s length and the strength of the applied magnetic field. We propose the use of COMSOL simulations and further exploration of the Abbasov model, alongside other relevant models, to guide the optimization of magnetic column design for these applications.

## Figures and Tables

**Table 1:** Summary of studies on label-Free and labeled magnetic separation of RBCs.

Type of Cell Detected	Label-free/Labeled	Aim of paper	Notes	Ref.
Malaria *(Plasmodium)* –infected red blood cells containing hemozoin (an Fe(III) paramagnetic complex)	Label-free	Compared the quantities of enriched RBCs and contaminating white blood cells of two density gradient centrifugation methods (Percoll and Nycodenz) and MACS column	Quantity of enriched RBCs was similar between MACS and centrifugation methods, but 90–99 % of white blood cells were removed with the MACS	[47]
		Capture of RBCs infected with *Plasmodium* at different stages, both *in vitro* and from patient blood samples	The first paper to utilize MACS for the enrichment of human non-falciparum *Plasmodium*	[48]
		Capture of *in vitro* Plasmodium-infected RBCs grown treated with sorbitol at different time intervals	Highest separation efficiency when schizonts predominated	[49]
		Capture of schizonts for erythrocyte invasion assays	Compared to the Percoll-sorbitol method, there was an improvement in the reproducibility and reliability of the invasion assay, especially for parasite isolates that cannot withstand exposure to the Percoll-sorbitol method	[50]
		Quantitative analysis of enrichment of *Plasmodium-* infected RBCs cultured *in vitro* with a saturation binding model	The purity of captured RBCs was influenced by the nature of the parasite and the density of infected RBCs. To improve capture efficiency, a lower flow rate and higher sample volume were suggested.	[51]
		Comparing the viability of the infected RBCs cultured *in vitro* enriched with either Percoll gradient centrifugation or MACS	MACS resulted in higher capture efficiency and viability of infected schizonts	[52]
		Comparing the gametocyte enrichment efficiency of standard centrifugation, Percoll gradient, and MACS in whole blood samples	MACS had the highest enrichment efficiency of gametocytes that could be assessed with mosquito-feeding assays for human infection studies	[53]
		Quantifying the percentage of gametocytes captured by the MACS columns and whether the columns can be reused without risk of contamination and lowering column binding efficiency	The enrichment rate of gametocytes was 94 % when the columns were used up to 5 times, with the rate decreasing to 79 % after eight reuses.	[54]
Maturing RBCs	Label-free	Enrichment of RBCs from hematopoietic stem cell-derived RBCS cultures and analyzing the magnetic mobility of the captured and eluted cells with CTV	The magnetically enriched fraction comprised 80 % of mature RBCs.	[55]
Nucleated fetal RBCs	Labeled (magnetic particles bound to specific antibodies)	Depletion of CD45+ and CD14- cells followed by the enrichment of CD71+ fetal RBCs in maternal blood samples	Known as the double MACS method with an average depletion rate of 780-fold and average enrichment rate of 500-fold	[56]
		Comparing the recovery of fetal RBCs of FACS with MACS from blood samples of women terminating their pregnancy	The yield and purity were significantly higher for FACS, but the specificity was significantly higher, and the cell loss was significantly lower for MACS	[57]
		Comparing the recovery of RBCs by using single or double gradient centrifugation in conjunction with MACS in samples of cord blood mixed with adult blood	Double-density gradient centrifugation led to a 68 % recovery of RBCs, but when combined with MACS, the purity improved by almost 200-fold.	[58]
		Triple-density centrifugation followed by CD71+ cell sorting and immunocytochemistry for the quantitative comparison of fetal RBCS from blood samples of mothers with and without intrauterine growth restriction	118.9 fetal RBCs per 10^5^ CD71+ cells in mothers with intrauterine growth restriction pregnancies compared to 11.5 cells in the control group	[59]
		Combination of centrifugation and MACS to separate fetal cells for the determination of the gender of the fetus	The specificity, sensitivity, negative and positive predicted value decreased with each trimester	[60]
		Comparison of one-step MACS (enrichment of CD71+ or GPA+ fetal RBCs) and two-step MACS (depletion of CD14+ maternal cells and enrichment of CD71+ or GPA+ cells) in 58 maternal blood samples	One-step MACS with the anti-GPA antibody was more effective than two-step MACS	[61]
		Comparison of one-step MACS (enrichment of CD71+ and GPA+ fetal RBCs) and two-step MACS (depletion of CD14+ maternal cells and enrichment of CD71+ and GPA+ cells) in 78 maternal blood samples obtained from 8–10 weeks and 11–14 weeks of gestation	The two-step MACS had a higher enrichment than the one-step MACS, but not significantly (p > 0.05) and there was no significant difference in the number of RBCs from 8–10 weeks and 11–14 weeks of gestation	[62]
		Capture of fetal RBCs with the anti-CD71 antibody and identification with Giema staining from the blood samples of 351 pregnant women	Identified fetuses that were normal and aneuploid and had trisomy 21 or β-thalassemia	[63]
Reticulocytes	Labeled	Compared four methods of capture: fluorescence-activated cell sorting (FACS), MACS, FACS+MACS, and Percoll gradient centrifugation from blood samples of sickle cell and healthy donors	All methods depleted reticulocytes to <0.1 % in healthy donor blood however Percoll gradient centrifugation depleted the reticulocytes to less than 4.8 and 2.5 ppm in healthy and sickle donor samples	[64]

**Table 2:** Analysis of variance (ANOVA) results of the process variable effects on the magnetic filtration efficiency.

Process variable	Domain	Log worth
Magnetic flux density	0.3,0.4 T	8.3
Particle size	3.5, 5, 6.5 μm	12.5
Flow rate	0.1, 0.2, 0.3, 0.4, 0.5 μl/min	12.5

**Table 3:** Abbasov model parameters from data fit to experimental RBC separations in a Miltenyi LS column with a 0.4 T external field.

Model parameter	Value	Units
$k_{1}$	−0.137	Unitless
$k_{2}$	−213	$10^{6}/mL$
$\beta^{\prime }$	0.0415	$s^{-1}$

## Data Availability

No data was used for the research described in the article.
